# Supplementary material for: Groundwater quality characterization using an integrated water quality index and multivariate statistical techniques
Source: PLoS One. 2024 Feb 23;19(2):e0294533. doi: 10.1371/journal.pone.0294533 (PMC10889601; doi:10.1371/journal.pone.0294533)
Supplement: Attachment — Submitted filename: Comments.docx [file pone.0294533.s001.docx]

**Author response to reviewer’s comments:**

Journal requirements:

When submitting your revision, we need you to address these additional requirements.

1. Please ensure that your manuscript meets PLOS ONE's style requirements, including those for file naming. The PLOS ONE style templates can be found at

<https://journals.plos.org/plosone/s/file?id=wjVg/PLOSOne_formatting_sample_main_body.pdf> and

<https://journals.plos.org/plosone/s/file?id=ba62/PLOSOne_formatting_sample_title_authors_affiliations.pdf>

**Reply: Yes, we have included as per suggestion.**

2. Please note that PLOS ONE has specific guidelines on code sharing for submissions in which author-generated code underpins the findings in the manuscript. In these cases, all author-generated code must be made available without restrictions upon publication of the work. Please review our guidelines at <https://journals.plos.org/plosone/s/materials-and-software-sharing#loc-sharing-code> and ensure that your code is shared in a way that follows best practice and facilitates reproducibility and reuse.

**Reply: Thanks for suggestion, no any code created by authors. All information shared in the article.**

3. Thank you for stating the following in the Acknowledgments Section of your manuscript:

“The authors extend their appreciation to Abdullah Alrushaid Chair for Earth Science Remote Sensing Research for funding.”

We note that you have provided funding information that is not currently declared in your Funding Statement. However, funding information should not appear in the Acknowledgments section or other areas of your manuscript. We will only publish funding information present in the Funding Statement section of the online submission form.

Please remove any funding-related text from the manuscript and let us know how you would like to update your Funding Statement. Currently, your Funding Statement reads as follows:

“No”

Please include your amended statements within your cover letter; we will change the online submission form on your behalf.

**Reply: Yes, funding statement is removed as per suggestion. Added in the Funded section.**

4. In your Data Availability statement, you have not specified where the minimal data set underlying the results described in your manuscript can be found. PLOS defines a study's minimal data set as the underlying data used to reach the conclusions drawn in the manuscript and any additional data required to replicate the reported study findings in their entirety. All PLOS journals require that the minimal data set be made fully available. For more information about our data policy, please see <http://journals.plos.org/plosone/s/data-availability>.

"Upon re-submitting your revised manuscript, please upload your study’s minimal underlying data set as either Supporting Information files or to a stable, public repository and include the relevant URLs, DOIs, or accession numbers within your revised cover letter. For a list of acceptable repositories, please see <http://journals.plos.org/plosone/s/data-availability#loc-recommended-repositories>. Any potentially identifying patient information must be fully anonymized.

Important: If there are ethical or legal restrictions to sharing your data publicly, please explain these restrictions in detail. Please see our guidelines for more information on what we consider unacceptable restrictions to publicly sharing data: <http://journals.plos.org/plosone/s/data-availability#loc-unacceptable-data-access-restrictions>. Note that it is not acceptable for the authors to be the sole named individuals responsible for ensuring data access.

We will update your Data Availability statement to reflect the information you provide in your cover letter.

**Reply: we have revised the data availability statement.**

5. Please include a caption for figure 6.

**Reply: Thanks for suggestions, we have included a caption of Fig.6.**

6. We note that Figures 1, 2 and 5 in your submission contain [map/satellite] images which may be copyrighted. All PLOS content is published under the Creative Commons Attribution License (CC BY 4.0), which means that the manuscript, images, and Supporting Information files will be freely available online, and any third party is permitted to access, download, copy, distribute, and use these materials in any way, even commercially, with proper attribution. For these reasons, we cannot publish previously copyrighted maps or satellite images created using proprietary data, such as Google software (Google Maps, Street View, and Earth). For more information, see our copyright guidelines: <http://journals.plos.org/plosone/s/licenses-and-copyright>.

We require you to either (1) present written permission from the copyright holder to publish these figures specifically under the CC BY 4.0 license, or (2) remove the figures from your submission:

1. You may seek permission from the original copyright holder of Figures 1, 2 and 5 to publish the content specifically under the CC BY 4.0 license.

We recommend that you contact the original copyright holder with the Content Permission Form (<http://journals.plos.org/plosone/s/file?id=7c09/content-permission-form.pdf>) and the following text:

“I request permission for the open-access journal PLOS ONE to publish XXX under the Creative Commons Attribution License (CCAL) CC BY 4.0 (<http://creativecommons.org/licenses/by/4.0/>). Please be aware that this license allows unrestricted use and distribution, even commercially, by third parties. Please reply and provide explicit written permission to publish XXX under a CC BY license and complete the attached form.”

Please upload the completed Content Permission Form or other proof of granted permissions as an "Other" file with your submission.

In the figure caption of the copyrighted figure, please include the following text: “Reprinted from [ref] under a CC BY license, with permission from [name of publisher], original copyright [original copyright year].”

2. If you are unable to obtain permission from the original copyright holder to publish these figures under the CC BY 4.0 license or if the copyright holder’s requirements are incompatible with the CC BY 4.0 license, please either i) remove the figure or ii) supply a replacement figure that complies with the CC BY 4.0 license. Please check copyright information on all replacement figures and update the figure caption with source information. If applicable, please specify in the figure caption text when a figure is similar but not identical to the original image and is therefore for illustrative purposes only.

The following resources for replacing copyrighted map figures may be helpful:

USGS National Map Viewer (public domain): <http://viewer.nationalmap.gov/viewer/>

The Gateway to Astronaut Photography of Earth (public domain): <http://eol.jsc.nasa.gov/sseop/clickmap/>

Maps at the CIA (public domain): <https://www.cia.gov/library/publications/the-world-factbook/index.html> and <https://www.cia.gov/library/publications/cia-maps-publications/index.html>

NASA Earth Observatory (public domain): <http://earthobservatory.nasa.gov/>

Landsat: <http://landsat.visibleearth.nasa.gov/>

USGS EROS (Earth Resources Observatory and Science (EROS) Center) (public domain): [http://eros.usgs.gov/#](http://eros.usgs.gov/)

Natural Earth (public domain): <http://www.naturalearthdata.com/>

**Reply: All the figures are prepared by the author itself. There is no copying from other sources. This is the PhD research work of the other, therefore all the map copy right reserved by the author. The author has already submitted this, on thesis.**

**Comments to the Author**

1. Is the manuscript technically sound, and do the data support the conclusions?

The manuscript must describe a technically sound piece of scientific research with data that supports the conclusions. Experiments must have been conducted rigorously, with appropriate controls, replication, and sample sizes. The conclusions must be drawn appropriately based on the data presented.

Reviewer #1: Yes

Reviewer #2: Yes

**Reply: Thanks for appreciated the work.**

2. Has the statistical analysis been performed appropriately and rigorously?

Reviewer #1: Yes

Reviewer #2: Yes

**Reply: Thanks for appreciated the work.**

3. Have the authors made all data underlying the findings in their manuscript fully available?

The [PLOS Data policy](http://www.plosone.org/static/policies.action#sharing) requires authors to make all data underlying the findings described in their manuscript fully available without restriction, with rare exception (please refer to the Data Availability Statement in the manuscript PDF file). The data should be provided as part of the manuscript or its supporting information, or deposited to a public repository. For example, in addition to summary statistics, the data points behind means, medians and variance measures should be available. If there are restrictions on publicly sharing data—e.g. participant privacy or use of data from a third party—those must be specified.

Reviewer #1: Yes

Reviewer #2: Yes

**Reply: Thanks for appreciated the work.**

4. Is the manuscript presented in an intelligible fashion and written in standard English?

PLOS ONE does not copyedit accepted manuscripts, so the language in submitted articles must be clear, correct, and unambiguous. Any typographical or grammatical errors should be corrected at revision, so please note any specific errors here.

Reviewer #1: Yes

Reviewer #2: Yes

**Reply: Thanks for appreciated the work.**

5. Review Comments to the Author

Please use the space provided to explain your answers to the questions above. You may also include additional comments for the author, including concerns about dual publication, research ethics, or publication ethics. (Please upload your review as an attachment if it exceeds 20,000 characters)

**Reviewer #1:**

The authors have presented a “Groundwater quality characterization with the integration of water quality index and multivariate statistical techniques”. Few correction and suggestion before it can be accepted.

**Reply: Thanks for suggestion and accepted the article for publication. As per you suggestion corrections were made in the revised article.**

1. How many year data was used for the study.

**Reply: Total 13 years of data have been used.**

2. Please indicate the novelty of the work.

**Reply: The study has been conducted in hard rock and tribal are of southern Rajasthan, which have more water quality problem as well as the water availability. Only the govt. agencies are working in this area, which is not sufficiently monitored. Therefore water quality was analyzed using previous data with multivariate statistical analysis and Geospatial technique, which is novel approach for these type of semi-arid hard rock region.**

3. Check Eq 2 for typo error.

**Reply: Corrected and improved**

4. Figure 6 is not stated in the text.

**Reply: added as per suggestion**

5. Figure 6 is not readable.

**Reply: These fig. are direct output of the analysis software. These are good quality graphs. Due to lower size, these have less visibility, but the size can be expanded as per requirement.**

6. Do these many figures are really needed or can you can add in supplementary data? Also remove the water mark from the figure 6.

**Reply: Yes, these graphs represents the mean, max and min value of the water quality parameters of the study area. Therefore it is necessary to present in main text of the manuscript.**

**Reviewer #2:**

The work by Gautam et al. deals with the characterization and interpretation of the groundwater quality (GWQ) using GIS environment and multivariate statistical approach (MSA) for Jakham River Basin (JRB) in the Southern Rajasthan. The topic is of great interest with the widespread application. Therefore, it is suggested for publication in the PLOS one journal, after a minor/moderate revision, which should include the following suggestions:

**Reply: Thanks for suggestion and accepted the article for publication. As per you suggestion corrections were made in the revised article.**

1- It would be beneficial to elaborate on the distinctive contributions of this research, especially in light of similar studies conducted previously. How does your work stand apart in terms of novelty?
**Reply: The study has been conducted in hard rock and tribal are of southern Rajasthan, which have more water quality problem as well as the water availability. Only the govt. agencies are working in this area, which is not sufficiently monitored. Therefore water quality was analyzed using previous data with multivariate statistical analysis and Geospatial technique, which is novel approach for these type of semi-arid hard rock region.**

2-How can the insights from this research be operationalized into tangible measures or technological solutions to enhance and safeguard groundwater quality?

**Reply: In the further recommendation of this research Decision support system are proposed for live detection and prediction of water quality in village level. Which will help to safeguard the potable water as well irrigation water.**

3-The decision to employ HCA over other advanced clustering techniques needs further justification. While its common use is acknowledged, the rationale for its selection in this specific context should be clear. Could you elaborate on this choice?

**Reply: As a data-driven method, HCA operates without a priori assumptions about aquifer geology, confinement, style and rate of water–rock interaction, or any other factors that might control the categorization.** **Another strength of HCA is that it can be based on any number of variables, and these variables can be of any type (chemical, physical, distributed or non-distributed). Therefore, in this study HCA was used as a cluster analysis tool.**

4-In determining the WQI using GIS, what criteria or parameters did you adopt to classify groundwater samples as 'good'?

**Reply: Total 13 parameters were identified for water quality indexing. Based on the analysis WQI value were determined for each observed well in the study area. The criteria based on the WQI single value given here: I (0-25)- Excellent, II (26-50)- Good, III (51-75)-Poor, IV (76-100)- Very Poor, V (> 100)- Unfit for consumption**

5-you should explain why the integrated approach of GIS and multivariate statistical approach (MSA) enhance the characterization of groundwater quality (GWQ) compared to using either method alone?

**Reply: multivariate statistical approach (MSA) helps to analyses the time series data of water quality, continuously these data can be applied as an input in the GIS tools for spatial mapping of groundwater quality. Therefore, this integrated technique is more precise than alone.**

6-The concluding section appears to have an abrupt ending. Kindly revise it.

**Reply: As per suggestion conclusion is revised.**

7-In conclusion section you should revise it based on the study's findings, what specific recommendations can be made for water resource planners and policymakers in the JRB?

**Reply: Based on the study, a consistent investigation of groundwater quality characteristics will reduce the prospect for further deterioration of soil and human health. However, the outcome of the present result may help to comprehend the excellence of the groundwater resources to improve appropriate management plan. Spatial map of WQI index will help to detect the live water quality for drinking as well irrigation purpose. Also the water health card can be generated based the predicted and present data. Added and improved as per suggestion.**

8-I noticed a potential issue in Equation 2; it appears there might be a missing parenthesis on the upper side. Could you please verify this?

**Reply: As per suggestion, equation have been improved and corrected.**

9- Figure 6 doesn't have caption. you must provide a detailed description for it.
Please double check the caption of figure 7 and 8 as well, you must mention the caption of each subplot as (a), and (b) separately.

**Reply: The caption provided in the fig.6. All the fig represents the box plot of water quality parameters, where (a) section displays pre monsoon data and (b) displays post monsoon season data. The details regarding graph already mentioned in the main text.**

6. PLOS authors have the option to publish the peer review history of their article ([what does this mean?](https://journals.plos.org/plosone/s/editorial-and-peer-review-process#loc-peer-review-history)). If published, this will include your full peer review and any attached files.


If you choose “no”, your identity will remain anonymous but your review may still be made public.


**Do you want your identity to be public for this peer review?** For information about this choice, including consent withdrawal, please see our [Privacy Policy](https://www.plos.org/privacy-policy).

Reviewer #1: No

Reviewer #2: No

**Reply: Thanks for suggestions.**
